# *Epilepsia Open*—February 2025 Announcements

**DOI:** 10.1002/epi4.13139

**Published:** 2025-02-07

## ILAE CONGRESSES

### ASEPA‐ANZAN Pre‐Congress EEG Teaching Course

February 18–19, 2025

New Delhi, India

### 15th Asian & Oceanian Epilepsy Congress

February 20–23, 2025

New Delhi, India

### 18th Latin American Summer School on Epilepsy

March 7–15, 2025

São Paulo, Brazil

### 1st Pediatrics Commission Symposium

March 21–24, 2025

São Paulo, Brazil

### 7th ILAE School on EEG in the First Year of Life

April 26–27, 2025

Sanya City, Hainan, China

### ILAE School on Neuroimaging 2025

May 15–17, 2025

Potsdam, Germany

### 5th African Epilepsy Congress

May 16–18, 2025

Lusaka, Zambia

### 15th ILAE School for Neuropathology and Neuroimaging in Epilepsy

July 31–August 3, 2025

Campinas, São Paulo, Brazil

### XVIII Workshop on Neurobiology of Epilepsy (WONOEP 2025)

August 25–29, 2025

Portugal

### 36th International Epilepsy Congress

August 30–September 3, 2025

Lisbon, Portugal

## 2026

### 16th European Epilepsy Congress

September 5–9, 2026

Athens, Greece

## ILAE Webinars

### ILAE Eastern Mediterranean & Africa webinaire

February 11, 2025

### ILAE Epilepsy and Autism

February 27, 2025

### ILAE Non‐convulsive status epilepticus

February 28, 2025

### ILAE ILAE e‐Forum: Diagnosis and classification of Developmental and Epileptic Encephalopathies (DEE)

April 22, 2025

## OTHER CONGRESSES

### 3rd International Online Course on Pathogenesis of Epilepsy

February 27–May 29, 2025

Online

### World Neuroscience and Psychiatry Conference 2025 (WNPC25)

March 8–9, 2025

Bangkok, Thailand

### 19th World Congress on Controversies in Neurology

March 20–22, 2025

Prague, Czech Republic

### 13th Trinational Conference of the German and Austrian Society for Epileptology and the Swiss Epilepsy League

March 26–29, 2025

Salzburg, Austria

### AD/PD™ 2025 International Conference on Alzheimer's and Parkinson's Diseases and related neurological disorders

April 1–5, 2025

Vienna, Austria & Online

### International Congress on Structural Epilepsy & Symptomatic Seizures 2025

April 2–4, 2025

Gothenburg, Sweden

### EPIPED Course: Treatment Strategies in Pediatric Epilepsies

23–26 April 2025

Girona, Spain

### 12th International Residential Course on Drug Resistant Epilepsies

May 11–27, 2025

Tagliacozzo, Italy

### International Conference for Technology and Analysis of Seizures (ICTALS 2025)

June 2–6, 2025

Montreal, Canada

### Korean Epilepsy Congress 2025

June 20–21, 2025

Seoul, South Korea

### 11th Congress of the European Academy of Neurology

June 21–25, 2025

Sevilla, Spain

### 21st San Servolo Advanced Epilepsy Course

21 July–1 August 2025

San Servolo (Venice), Italy

### 19th European Congress for Clinical Neurophysiology

September 9–12, 2025

London, UK

### 19th Panhellenic Congress of Epilepsy

September 25–28, 2025

Heraklion, Crete, Greece

## 2026

### 18th Eilat Conference on New Antiepileptic Drugs and Devices

May 3–6, 2026

Madrid, Spain

